# Intersection of scalp melanoma and nurofibromatosis type 1 rare case report

**DOI:** 10.1016/j.ijscr.2023.108186

**Published:** 2023-04-13

**Authors:** Abdullah Rahamah, Khalid Elsir Idris, Jok Kuol Monyluak Dak, Mohammed Hatim Hamdto

**Affiliations:** a Omdurman Teaching Hospital, Sudan; b Faculty of Medicine and Health Sciences, Omdurman Islamic University, Sudan

**Keywords:** Neurofibromatosis, Melanoma, Case report

## Abstract

**Introduction and importance:**

Type 1 neurofibromatosis is a multisystem disease, mainly involving the nervous system and skin. Complications of type 1 neurofibromatosis are the development of neural crest derived malignancies such as melanoma of the skin. Skin melanomas have been found in 0.1 %–5.4 % of NF1 patients and it's far less likely to develop in Black people.

**Case presentation:**

We present a 53 years old female known to be a case of NF-1 with multiple neurofibromas. One of them in the scalp since the age of 12. Two months prior to presentation, the scalp neurofibroma start to increase in size rapidly and multiple ulcers developed. CT scan showed soft tissue mass not attached to the bone or other cranial structure. The patient underwent excision with safety margins with a full thickness skin graft. Histopathology showed a free edge of the tumor and was initially suggestive of spindle cell sarcoma then immune-histo-chemistry requested the result showed, melanoma of the scalp.

The follow-up showed a good take of skin graft and the patients was sent to oncology for further management.

**Clinical discussion:**

Melanoma is a rare malignancy in patients with NF1, but it can occur. It is important to be aware of the possibility of melanoma in these patients and to perform regular skin checks. Treatment of melanoma in NF1 patients follows the same guidelines as for non-NF1 patients, with wide or narrow excision margins depending on the type of melanoma. Reconstruction of the defect can be done with split-thickness or full-thickness skin grafts, depending on the size and location of the defect.

**Conclusion:**

Melanoma is a devastating skin malignancy and should be in suspicion for any skin lesion that present even in association with rare diseases.

## Highlights

• Neurofibromatosis is an autosomal dominant disease associated with multisystem involvement mainly skin and nervous system
• Neurofibromatosis is associated with increased risk in comparison with the general population.
• 53 year old female diagnosed with Type 1 Neurofibromatosis (NF-1)
• Patient had a neurofibroma on the scalp since age 12 Neurofibroma started to increase in size rapidly and multiple ulcers developed
• CT scan showed soft tissue mass not attached to bone or underlying cranial structure
• Patient underwent excision with safety margins and full thickness skin graft
• Histopathology initially suggestive of spindle cell sarcoma Immunohistochemistry revealed melanoma of the scalp

## 1 Introduction

Type 1 neurofibromatosis is a multisystem disease, mainly involving the nervous system and skin. With variability in clinical manifestations even within one family [1]. With an occurrence of approximately 1 in 3000 births, von Recklinghausen type 1 neurofibromatosis is considered one of the most common inherited disorders considered as neurocristopathy, a disorder of neural crest-derived cells. One of the complications of type 1 neurofibromatosis is the development of neural crest-derived malignancies such as malignant schwannoma, pheochromocytoma, and malignant melanoma of the skin and choroid [2] von Recklinghausen disease is the result of mutations of the NF1gene which encodes a protein called neurofibromin, which negatively regulates the Ras-dependent pathway. An excess of tumors especially tumors of a neuroectodermal source is usually observed [3]. Neurofibromatosis patients have more risk of developing neoplasms when compared to general population malignant peripheral nerve sheath tumors (MPNST) are the most common neoplasms. Patients with NF1 are at risk to melanoma, compared with the general population [4]. Skin melanomas have been found in 0.1 %–5.4 % of NF1 patients [5]. Black people are far less likely to develop melanoma than non-Hispanic White people (at a rate of 1 per 100,000 compared to 30 per 100,000) due to the protection that melanin provide [6]. We are here to present a case of melanoma of the scalp that was extremely challenging to diagnosis.

SCARE checklist was used in this report [7].

## 2 Presentation of case

**Fig. 1:**
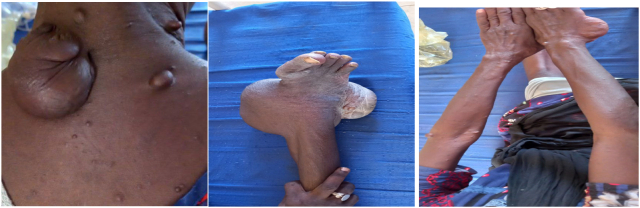
Multiple neurofibromas of the patient.

A 53-year old female known to be a case of NF-1 was presented to us due to rapid progressive growth of scalp mass on top of scalp neuro-fibroma causing the patient to complain of headache, heaviness sensation, and dizziness. The patient has scalp lump since the age of 12 which was static in size. Two months prior to presentation. The scalp lump begins progressively to increase in size to become a large fungating mass with multiple ulcers. The patient has multiple neuro-fibroma including multiple small ones in the back and upper limbs large one in the neck and right heel. The total of neuro-fibromas was more than 23 (Fig. 1).

**Fig. 2:**
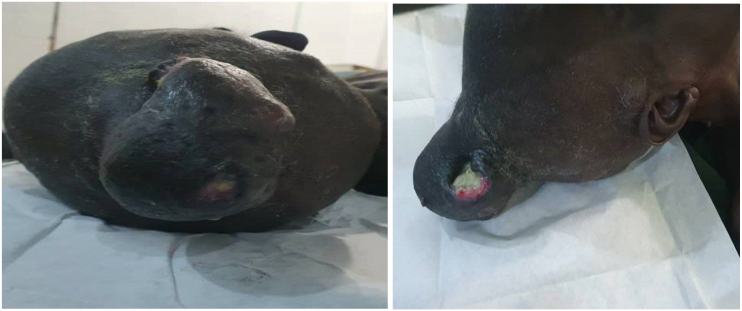
Show scalp neurofibroma lesion.

The clinical examination revealed a large lump of 6 × 8 × 6 cm in the occipito-parietal region firm in consistency, well defined edges, with multiple ulcers on the surface, no palpable cervical lymph node (Fig. 2). Otherwise systemic examination was unremarkable.

**Fig. 3:**
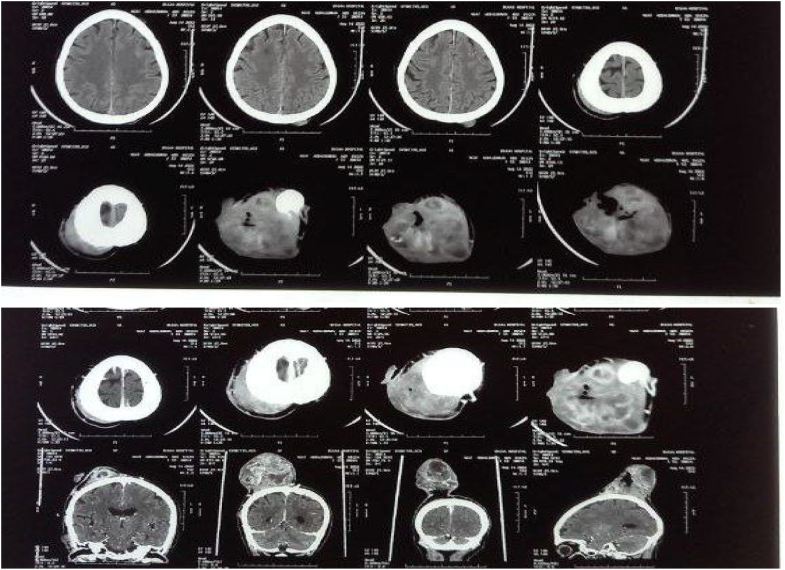
CT scan show will define heterogeneous subcutaneous masses with no evidence of underlying bone destruction, invasion and intracranial lesion or invasion.

**Fig. 4:**
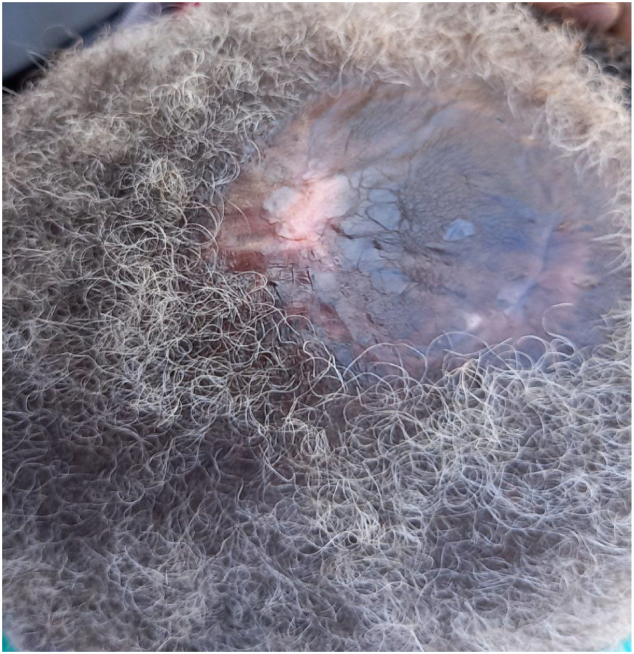
Show outcome after 6 weeks.

Investigation reviled normal hematological findings with hemoglobin 11.4 g/dl total white blood cell of 8.4 × 109/L and platelet 461 × 10^9^/L normal renal function, electrolyte and normal random blood glucose. Chest x-ray and ECG were also performed and were normal. The skull x-ray didn't reveal any abnormality. CT brain with contrast was done and showed will define heterogeneous subcutaneous masses with no evidence of underlying bone destruction, invasion, or intracranial lesion (Fig. 3). Fine needle aspirations were performed and showed atypical cells putting in suspicion of sarcoma. The patient was prepared and underwent tumor excision with a safety margin and full-thickness skin graft under general anesthesia the patient received 1 unit of blood intra-operatively. The operation went smoothly excision of the tumor was done with a 3 cm safety margin with an intact underlying gallia. The defect was covered with full-thickness skin graft from the right groin the donor site was closed primarily. The excised tumor sent for histopathology and the patient was discharged from hospital without any complication at day 1 postoperatively. First dressing was done after 5 days from operation showing good take of the graft the histopathology were obtained showing infiltrative malignant composed of plump spindle cell with mild pleomorphic, and increased mitotic activity. The surgical margins were free of tumor. Histopathology findings were in favor of spindle cell sarcoma and immune histo- chemistry was requested for further confirmation. One month form the operation the patient come to follow up the skin graft showed good take yet there was some dryness the patient was advised to apply ointment. The result of the immune-histo-chemistry relived S-100 diffusely positive SMA are negative control is positive and CK negative this excludes spindle cell sarcoma, suggestive that the scalp mass was malignant melanoma. The patient was referred for oncology for further management. Fig. 4 shows the final outcome 6 weeks post-operative showing good take of the skin graft.

## 3 Discussion

Patients with NF1 are usually more susceptible to malignancies compared to the general population, the commonest is MPNST [4]. Cutaneous melanomas were reported in many cases with multiple locations in the body including the penis and back [3], [7]. We presented a rare case of malignant melanoma in Sudanese patient with long-standing scalp neurofibromas which was challenging to diagnosis. Neurofibroma is as common in Black as it's in the White population yet melanoma is not as much common, in Black as it's in White. In most of the reported cases in literature, melanoma arises from pigmented nevus on different location. Nasir, Wasim et al. described melanoma arising on top of neurofibroma without a pervious diagnosis of neurofibromatosis [8]. The association between the two diseases can be explained by RAS mutations described in patients with NF-1 this mutations also recognized in patients with melanoma described as unholy [9], which may explain the development of melanoma in patient with NF-1. Management of scalp melanoma follows the same rule as non-head and neck melanoma with wide 5 cm or narrow 3 cm safety margin [10], [11]. Most excisional defects of the scalp melanomas are reconstructed with split thickness skin grafts, rarely flap coverage is required [11]. Although the previous studies suggested that the patient must underwent split-thickness skin graft we chose full thickness to provide adequate cover, also the full thickness graft was easier to harvest in presence of multiply scattered neurofibromas all over to body reducing morbidity and facilitated its closure primarily.

## 4 Conclusion

Melanoma is a devastating malignancy that should be put under suspicion in any patient presented with a skin tumor. Although it is rare, it can be associated with other rare clinical syndromes like neurofibromatosis type one.

## Consent to publish

Written informed consent was obtained from the patient for publication of this case report and the accompanying images.

**Figure:**
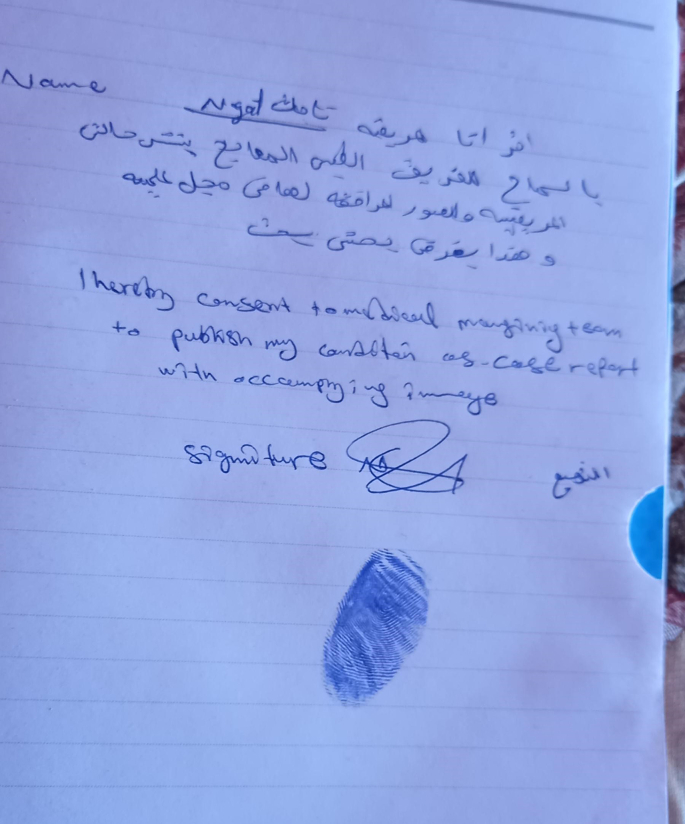

## Ethical approval

The case report were performed following declaration of Helsinki and according to international guideline and ethics.

## Funding

None.

## Guarantor

Khalid Elsir Ahmed.

## CRediT authorship contribution statement

Abdullah Rahama generation of study concepts, revision of the report
Khalid Elsir writing the report draft, writing the final report and the revision
Jok Kuol Monyluak Dak writing the final draft, revision
Mohammed Hatim Hamdto revision and final draft.

## Conflict of interest

None.
